# Spray dried VSV-vectored vaccine is thermally stable and immunologically active in vivo

**DOI:** 10.1038/s41598-020-70325-2

**Published:** 2020-08-07

**Authors:** Steven P. Toniolo, Sam Afkhami, Michael R. D’Agostino, Brian D. Lichty, Emily D. Cranston, Zhou Xing, Michael R. Thompson

**Affiliations:** 1 Department of Chemical Engineering, McMaster University, Hamilton, ON Canada; 2 McMaster Immunology Research Center, McMaster University, Hamilton, ON Canada; 3 Department of Pathology & Molecular Medicine, McMaster University, Hamilton, ON Canada; 4 Department of Wood Science, The University of British Columbia, Vancouver, Canada; 5 Department of Chemical and Biological Engineering, The University of British Columbia, Vancouver, Canada

**Keywords:** Biochemistry, Drug discovery, Drug development, Chemical engineering, Infectious diseases, Viral infection

## Abstract

Effective vaccine delivery and coverage to rural and resource-poor countries is hindered by the dependence on cold chain storage. As such, developments of cold chain-free technologies are highly sought. Although spray dried adenoviral vectors have shown long term stability at ambient temperatures and relatively low humidity, it remains to be determined whether similar excipient formulations are applicable to other viral vectors. To address this, we have spray dried vesicular stomatitis virus (VSV)-vectors with a panel of well-characterized sugar excipients to determine the optimal formulation for vector stabilization. Upon reconstitution, we show that trehalose conferred superior stability of VSV both in vitro and in vivo. Importantly, following cold chain-free storage at elevated temperatures at 37 °C for 15 days, we show that a VSV-vectored vaccine retains its in vivo immunogenicity, whereas a liquid control completely lost its immune-stimulating ability. Our results provide foundational evidence that spray drying with properly tested excipients can stabilize viral vectors such as VSV, allowing them to be stored long-term at elevated temperatures without dependency on cold chain conditions.

## Introduction

Vesicular stomatitis virus (VSV), an enveloped and single-stranded RNA virus, has become a choice of vaccine vector for infectious diseases and cancer^1,2^. Clinical evidence supporting the utility of VSV-vectored vaccines has been recently documented with success in the fight against Ebola, including the latest Ebola outbreaks in Africa^3^.

VSV-vectored vaccines are normally stored in aqueous media and therefore must be refrigerated to maintain their long-term efficacy^4,5^. As a result, cold chain protocols are implemented to retain viral activity during transportation and storage. This impedes the ability to stockpile large quantities of vaccines, thereby limiting their distribution to resource-poor areas where cold chain requirements cannot be feasibly met^6^. The World Health Organization (WHO) has advocated for a controlled temperature chain (CTC) permitting vaccines to be exposed outside of the cold chain temperature (exceeding 2–8 °C) for short periods of time^7^. For CTC approval, vaccines must retain their efficacy after being stored at temperatures in excess of 40 °C for a minimum of 3 days^7^. To mitigate the need for cold chain storage of liquid vaccines, research continues to be directed towards developing thermally stable vaccines, through the vitrification of excipients encapsulating these biologics into glassy powders^8^. This stability does not eliminate the need for cooler transport/storage conditions, especially in hot climates, but eases the technology demands and extends the length of time that the cold chain can be broken before the vaccine needs to be discarded. Efforts to vitrify adenoviral- and influenza viral-based vaccine vectors with saccharide-based excipients have been successful, demonstrating thermal stability for prolonged periods of time when stored in this dried state^9,10^.

For industrial applications, lyophilisation has often been selected as the preferred method of drying biologics^11^. However, deficiencies with the lyophilisation process exist. Many biologics are susceptible to the physical and chemical stresses that occur during freezing and drying processes^12^, and the batch process of lyophilisation is extremely time-consuming and limiting in throughput. In comparison, spray drying has become a promising approach which addresses the deficiencies associated with lyophilization and is capable of generating thermally stable vaccines which retain their efficacy^5^. Briefly, spray drying uses pressurized gas to aerosolize a liquid feed containing the biologic and excipients into a chamber containing heated air. The liquid droplets rapidly evaporate, forming a dry powder collected in a cyclone unit. The ability to tune characteristics of the dried particles such as particle size, degree of crystallinity, and density, as well as the scalability of the process and short batch time have made spray drying an attractive alternative to lyophilization. The spray drying of powdered vaccines against measles, tuberculosis, hepatitis B, and influenza for example, has been explored with demonstrated improvements in thermostability^9,13–15^.

While there has been some notable success in achieving thermal stability of viral-based vaccines such as influenza and adenoviral-based vectors via spray drying, it cannot be assumed that all viral vectors will behave similarly upon vitrification. There is an inherent need to address the underlying phenomenon that not all excipients encapsulate the various families of viruses similarly^16^. Thus, the objective of our current study was to first investigate the effects of various excipients on the thermal stability of a spray dried VSV viral vector in vitro and to subsequently assess the immunogenicity of selected spray dried VSV vector in vivo. Through spray drying of the model VSV vector expressing green fluorescent protein (VSVGFP), we have identified excipients that minimize the loss of viral activity resulting from the spray drying process. Additionally, the spray dried powders encapsulating VSV were subjected to varying storage conditions to investigate which excipients provided the best thermal stability to the viral vector. To address any possible defects in the viral structure that may hinder in vivo immunogenicity, a spray dried VSV vector expressing a model *M. tuberculosis* antigen, Ag85A, (VSVAg85A) was similarly aged and tested in mice. The goal of this work was to highlight which excipients perform best with the VSV vector, such that the excipients minimize activity loss during the spray drying process and maximize thermal stability over long-term storage, thereby reducing the dependency on cold chain storage. Relatively high activity losses are reported due to the non-optimal spray drying conditions needed to compare numerous excipients under comparable process stresses to the viral vector, and so conclusions of performance are drawn based on trends only; optimized systems should be reasonably expected to show no more than 0.5 log loss under CTC evaluation (based on past optimization work in our group^17,18^), though this is more of an industrial value than a regulatory specification.

## Results

### Thermal stability of spray dried VSV vectors is excipient-dependent

**Figure 1:**
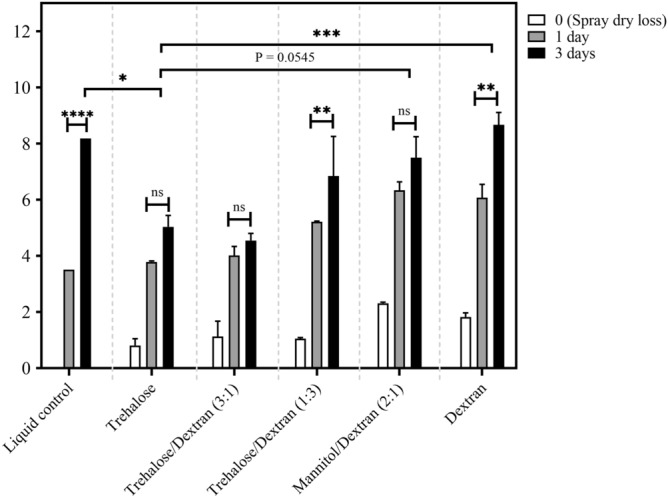
Measured log loss of VSVGFP activity after storage at 45 °C and < 10% RH for the positive (liquid) control, trehalose, trehalose/dextran 3:1, trehalose/dextran 1:3, mannitol/dextran 2:1, and dextran. Process loss attributed to spray drying is shown at t = 0 (spray dry production loss) and loss after 1 and 3 days of storage is compared. Data are represented as mean ± SEM from repeats for all formulations. The results of a two-way ANOVA between time points for each excipient formulation are shown where *P = 0.05, **P = 0.001, ***P = 0.0002, ****P < 0.0001, ns: not significant.

We have previously characterized numerous excipients which show a range of stabilization properties when used to spray dry a human adenovirus serotype 5 (AdHu5)-vectored vaccine^16^; the selected excipient candidates were chosen for their suitability for vaccine administration. We first sought to profile excipients that best stabilize VSV by utilizing an in vitro endpoint dilution assay to assess the infectivity of a VSV vector expressing GFP (VSVGFP); stability trends found with the excipients for the viral vector are expected to be reflected with VSVAg85A (as shown previously for AdHu5GFP vs. AdHu5Ag85A^19^) but the scope of the in vitro studies would have been severely limited without the relatively rapid quantification of viral activity based on detection of a fluorescent response. To this end, VSVGFP was spray dried utilizing different excipients under spray drying conditions detailed in the methods section. All spray dried powders were produced under similar spray drying conditions. The storage conditions used in the study were selected to exceed CTC requirements stated by the WHO, which require the viral vector to demonstrate activity after storage in excess of 40 °C for a minimum of 3 days^7^. The activity log losses for considered single and blends of excipients after the spray drying process (Day 0) and over 3 days stored dry at 45 °C along with their respective excipient composition (ratios by mass) are shown in Fig. 1.

Trehalose and dextran, as single encapsulating ingredients, and all excipient blends, showed acceptable activity losses between 0.8 and 2.6 log (production loss, Day 0). In contrast, mannitol exhibited a high activity loss of 4.8 (data not shown) and was not investigated further. When spray dried samples were stored at 45 °C for one day, however, differences in the stabilization properties of the different excipients became more apparent (Fig. 1). For example, the mannitol/dextran blend that initially appeared as an acceptable stabilizer at Day 0 was now showing poor performance with high activity losses on Day 1. This is very different stabilization behaviour with VSV compared to our previously published work with human adenovirus serotype 5 vectors, which found superior stabilization with mannitol/dextran blends^9,19^; an initially tested 3:1 mannitol/dextran blend (data not shown) performed even worse, exhibiting a loss of 8.3 log by Day 1, and was removed from the study. It has been postulated that different groups of excipients are required to stabilize viral vectors depending on whether they possess an envelope^16^.

In comparison to these results with mannitol-based formulations, trehalose and trehalose blends showed good stabilization properties for VSV after Day 1 (Fig. 1). Trehalose as a single encapsulating ingredient and in blends of 3:1 with dextran performed equally well with losses of 3.8–4.0 log. Only the 1:3 trehalose/dextran blend showed higher activity losses of 5.2 on Day 1. It seemed that high concentrations of dextran were undesirable with VSV.

After 3 days of storage at 45 °C, the stability of the better excipient formulations noted on Day 1 became even more apparent, especially compared to the positive liquid control that exhibited near-negligible activity with a reported log loss of 8.2 (Fig. 1). Particles with trehalose or the blend of trehalose/dextran (3:1) showed a slower rate of activity losses with VSV, log loss of 4.9 and 4.5, respectively, compared to the other excipient formulations, with closer to 7–9 log loss (Fig. 1). The less-than-optimal blends noted for Day 1 of mannitol/dextran (2:1) or trehalose/dextran (1:3) continued to perform poorly on Day 3 and only slightly better than the positive control or dextran alone.

Statistical analysis on Fig. 1 data indicates that overall, the increased activity loss for all formulations is significant between Day 0 and Day 1 (analysis performed but not shown on plot for clarity), but is only significant between Day 1 and Day 3 for the liquid control, trehalose/dextran (1:3), and dextran. Comparing between formulations at Day 3 supports that trehalose is significantly better at thermally stabilizing VSV compared to the control, mannitol/dextran (2:1) and dextran but is similar to both trehalose/dextran formulations.

**Figure 2:**
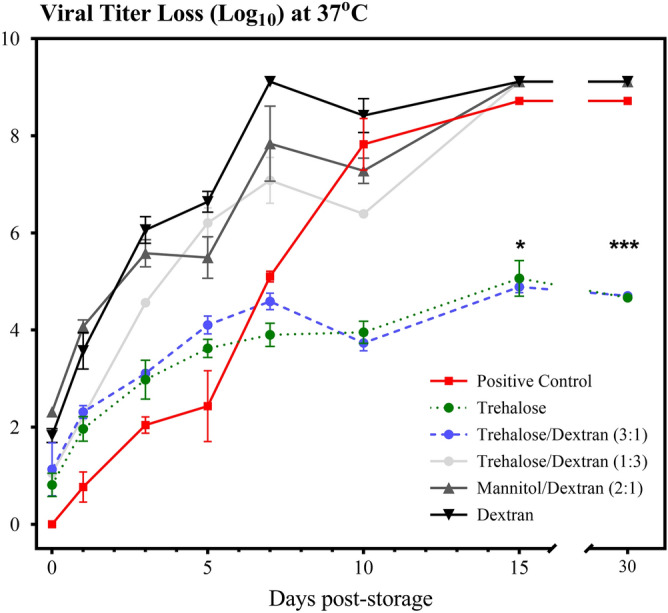
Measured log loss of VSVGFP infectivity after storage at 37 °C and < 10% RH for the positive (liquid) control, trehalose, trehalose/dextran 3:1, trehalose/dextran 1:3, mannitol/dextran 2:1 and dextran. Data are represented as mean ± SEM for three repeat samples; all data points have error bars but in some cases they are indistinguishable from the symbol itself. The results of a two-way ANOVA between excipient formulations at day 15 and 30 are shown where *P = 0.05, ***P = 0.0002, indicating that trehalose and trehalose/dextran 3:1 significantly outperform the other formulations and the liquid control at end of the storage study.

The testing at 45 °C was too harsh for prolonged studies longer than three days but it was apparent that preferred formulations for stabilization were more apparent as storage time increased. To further dissect the stabilization properties of these excipients for the VSVGFP vector, the in vitro studies were extended for up to 30 days of storage but conducted at a lower temperature in order to observe differences over the whole duration. The results in Fig. 2 corresponded to the physiological temperature of 37 °C. With the more protracted length of storage, the displayed trends confirm the findings at 45 °C that trehalose and trehalose/dextran (3:1) were statistically the best performing excipient formulations, showing lower activity losses of the VSVGFP vector (4.8 log loss for these preferred formulations rather than approximately a log loss of 9 for the other formulations, Fig. 2). After 7 days of storage at 37 °C, the activity loss of the positive control was much greater than trehalose and trehalose/dextran samples, and all activity of the positive control was lost by Day 15. After 10 days of storage, the activity loss of trehalose and trehalose/dextran (3:1) appeared to reach a plateau. The mannitol/dextran blend demonstrated the most activity loss initially and over time, and was deemed to be the worst performing excipient blend for thermally stabilizing VSVGFP.

### Evaluation of immunogenicity in vivo

While our in vitro GFP assay provides insight on the retention of viral activity in spray dried VSV vectors after storage, it is relevant to determine if the findings are translatable in vivo. Based on our in vitro storage data showing that trehalose was the best at thermally stabilizing VSV after spray drying, we chose this excipient to assess the in vivo immunogenicity of our spray dried vaccine in a mouse model. To do so, we opted to utilize a VSV vector expressing the mycobacterial antigen Ag85A^20^. As this vector works the best when administered as a heterologous booster^1,20^, we initially primed all animals with a human adenovirus serotype 5 vector expressing the same antigen Ag85A (AdHu5Ag85A). Thus, the mice were first primed intramuscularly (I.M.) with AdHu5Ag85A. Two weeks post-priming, they were boosted I.M. with either a liquid control of VSVAg85A, or trehalose spray dried samples (reconstituted in PBS), immediately after spray drying (d0), or samples stored for 7 (d7) or 15 (d15) days at 37 °C. A subset of primed animals was not boosted as controls for the baseline (prime) immune response. Two weeks post-boosting, animals were sacrificed and Ag85A-specific immune responses were assessed in the spleen via Ag85A tetramer immunostaining. Knowing that the VSVAg85A vector is weakly immunogenic when used as a stand-alone vaccine, coupled with the large activity losses seen in vitro after spray drying and storage supported the use of an Ad prime-VSV boost regimen to more sensitively detect VSV-induced immune activities.

**Figure 3:**
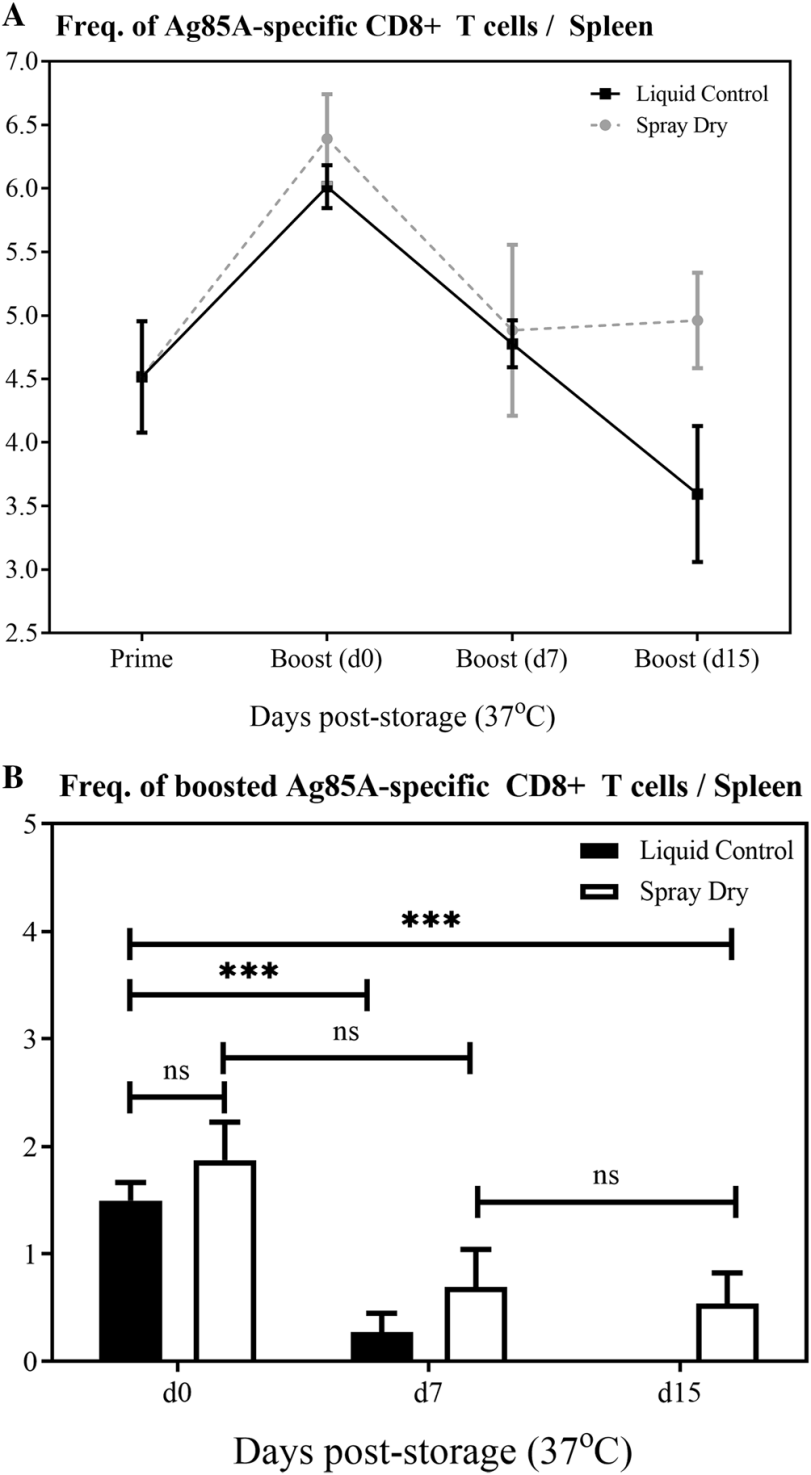
In vivo immunogenicity of trehalose spray-dried VSVAg85A following storage at 37 °C. Animals were I.M. prime-immunized with AdHu5Ag85A and were I.M boosted with VSVAg85A 2 weeks post-priming. Animals were subsequently sacrificed 2 weeks post-boost and Ag85A-specific CD8 T cell responses were assessed via tetramer immunostaining. A subset of primed animals was not boosted (prime only) or were boosted with either the liquid control of VSVAg85A or trehalose spray-dried equivalent, which were stored for 0, 7, and 15 days at 37 °C, (**a**) shows the total frequency of Ag85A-specific CD8 T cell responses whereas (**b**) shows only the boosted immune response (boosted response = total response minus the ‘prime only’ response). Data are expressed as average values ± SEM of 3 animals per group and unpaired T-test results with ***P = 0.0002 and ns: not significant, are shown.

In agreement with our previous observation^1^, VSVAg85A boosted Ag85A-specific CD8 T cell responses to levels greater than that seen in animals just primed with AdHu5Ag85A, with the spray dried vector and liquid control immune responses being equivalent before ageing (Fig. 3, Day 0 boost). As with our in vitro storage studies where minimal activity loss was seen 7 days post-storage at 37 °C, we noted a small drop in the Ag85A-boosting capacity of VSVAg85A, similar to the liquid control (Fig. 3a). However, when stored for 15 days in similar conditions, the spray dried VSV vector retained its immunogenicity, showing statistically similar boosting capacity as samples stored for 0 and 7 days (Fig. 3b). In stark contrast, the boosting capacity of the liquid control was completed ablated, (with a significant loss in its boosting capacity of Ag85A-specific immune responses at each storage time point) ending with complete loss of viral activity following 15 day-storage at 37 °C.

## Discussion

The purpose of this study was to demonstrate that VSV-vectored vaccines can be spray dried with suitable excipients to retain thermal stability in a powder form and maintain activity in both in vitro and in vivo tests after prolonged storage. Previous work has established that spray drying is a viable method to thermally stabilize influenza and adenoviral vectors^5,9^. In another recent study, we have also shown spray drying to be a viable method for increased thermostability of VSV vectors but examined the chemical interactions with the stabilizing excipients rather than considering the prolonged storage and in vivo application^16^. The current work highlights that trehalose and a blend of trehalose/dextran at a 3:1 weight ratio, spray dried from a 4 wt% solution, were both able to thermally stabilize the VSV vaccine vector and retain its activity significantly better over time compared to the liquid control. This represents a significant improvement compared to the generally prescribed cold chain storage requirements for vaccines based on VSV.

The role of trehalose in the improved stabilization of the VSV vector is likely based on its ability to replace the water molecules typically surrounding the lipid membrane envelope of the virus. It is understood that, upon dehydration without added excipients, the viral lipid envelope becomes rigid as the hydrogen bonds to water are removed, and an increase in van der Waals forces leads to the hydrophobic tails of the lipid bilayers aggregating and becoming transiently leaky^21^. When rehydrated, the lipid membrane experiences localized rigidity leading to the leakage of inner viral components through gaps in the membrane^21^. To effectively stabilize lipid membranes in a dry state, the hydrogen bonds that exist between water molecules and the lipid phosphate heads must be substituted. The *water replacement hypothesis* suggests that the hydroxyl groups of carbohydrates can replace the main hydration shell surrounding the polar phosphate head of the lipid membrane of the enveloped virus^22^.

Trehalose has shown itself to be an excellent carbohydrate in stabilizing lipid membranes of biologics in a dehydrated state^23^ and has been used to stabilize enveloped viruses, such as influenza vectors, in the dry state^24^. The hydroxyl groups of trehalose form direct hydrogen bonds with the phosphate heads of the lipid bilayer, effectively replacing the removed water molecules^16^. Additionally, trehalose is able to form a primarily amorphous matrix to encapsulate and protect the viral vectors from external stresses and fusion of the lipid membranes which would otherwise lead to leakage^22^. However, the extent to which this carbohydrate is used in formulations should be optimized for the duration of anticipated storage, since it is more moisture sensitive than the other excipients examined. Even under the low humidity conditions used for storage at 37 °C, the reported glass transition temperature values for trehalose and trehalose/dextran (3:1) declined from 110 to 88 °C, respectively for Day 0, to 58 °C for Day 15 whereas mannitol/dextran only declined from 113 to 99 °C over the same duration^16^. Due to the branched nature of dextran it tends to be fairly amorphous, but the poor performance observed for the spray dried dextran-only sample is likely due to steric hindrance. Unlike other, smaller sugars, the larger size of dextran was unable to replace the hydrogen bonds formed between the water molecules and individual phosphate heads of the lipid membrane as effectively^24^. The blend of trehalose/dextran (3:1), was successful due to the relatively large amount of trehalose that could be used to replace the hydrogen bonds; the robustness of this blend to stabilize VSV vaccines lacking the wild-type glycoprotein would require additional study though it is anticipated that these two carbohydrates would remain suitable possibly in a different ratio. Furthermore, the spray dried mannitol/dextran blend, which demonstrated exceptional thermal stability with adenoviral vectors^9^, was the worst performing excipient blend when stabilizing the VSV vector. This is likely due to the high tendency of mannitol to crystallize, which is particularly damaging to enveloped viruses, leading to membrane rupture and leakage of inner viral components.

Our in vivo study further supports the conclusion that spray drying with an optimal formulation conditions can stabilize VSV vectors in such a way that they can be stored for prolonged periods of time at-or-above physiological temperatures without losing immunogenicity. While trehalose, the best performing excipient, experienced a loss in vitro of greater than 4 log over 30 days at 37 °C, it is imperative to note that the spray drying parameters for all excipient formulations were not optimized so that they could all be compared under similar thermal and shear stresses. Parameters such as initial excipient concentration, inlet spray dry temperature and spray gas rate can be adjusted in the future to achieve optimal conditions for minimized activity losses. Thus, it is likely that further reductions in activity loss may be possible and would better meet the WHO guidelines for CTC.

## Methods

### Chemicals and VSV vectors

d-(+)-Trehalose dihydrate, d-mannitol, and dextran (M_r_ 40,000 kDa) were purchased from Sigma-Aldrich (Ontario, Canada). Recombinant Vesicular Stomatitis Virus-based vaccine vectors (VSVAg85A and VSVGFP) were produced in the vector facility of McMaster Immunology Research Centre as described previously^1^. The vaccine vector is replication-competent containing the VSV wild-type glycoprotein.

### Spray drying of VSV-vectored vaccines

Spray dried powder viral vectors were produced using a Mini Spray Dryer B-290 (Büchi; Switzerland) with 0.7 mm spray nozzle and high performance cyclone attachments as previously described^10^. For all spray dried excipients, the spray dryer was operated at a spray gas flow rate of 439 L/h, feed solution of 234 mL/h and a nozzle inlet temperature of 110 °C. The spray dried formulations were selected through preliminary testing or based on previous work^9^.

### Vaccine sample storage

Powder samples were stored as previously described^10^. Briefly, powders were enclosed in 2 mL Nalgene cyrogenic tubes and sealed with parafilm wax. Tubes were stored within a glass jar which were lined with gel desiccant, ensuring low humidity (< 10% relative). Glass jars were submerged within a temperature-controlled water bath at the desired experimental temperature. Liquid controls were stored in a similar fashion.

### Culturing of vero cells

Vero cells (ATCC CCL-81) isolated from kidney epithelial cells were thawed from liquid nitrogen and cultured with Alpha Minimum Essential Medium Eagle (α-MEM) supplemented with 10% FBS, 1% l-glutamine, and 1% Penicillin/Streptomycin in the same fashion as previously described^10^. Assays were preformed by seeding cells into 96-well flat bottom plates, in accordance with the desired experimental requirements.

### Spray dried viral infectivity

Viral activity following the spray drying process and subsequent storage was determined by an endpoint dilution assay as previously described^10^. Briefly, Vero cells were infected with serially-diluted cell media-reconstituted powder (initial concentration of 1.30 $\times$ 10^9^ PFU/mL) and left to incubate overnight. Following overnight incubation, viral infectivity was detected by the presence of Green Fluorescent Protein (GFP) expression under an EVOS FL Cell Imaging System (Thermo Scientific Corporation; Waltham, MA). A positive GFP response of a single cell within a well constituted a positive infection response with respect to the endpoint dilution. If greater than 50% of the wells in the row were non-expressive, the dilution was determined to have reached its endpoint and the median tissue culture infections dose was calculated using the Reed-Muench method. Two-way ANOVA multiple comparisons were done to compare between time points for each excipient (number of families = 6 (excipients), number of comparisons per family = 3 (timepoints), where *P = 0.05, **P = 0.001, ***P = 0.0002, ****P < 0.0001, and ns: not significant).

### In vivo evaluation of immunogenicity of spray dried vaccine

Female BALB/c mice, 6–8 weeks old, were purchased from The Jackson Laboratory (Bar Harbor, ME) and housed in a specific pathogen-free level B facility at McMaster University. All animal experiments and experimental protocols pertaining to animal handling and use were approved by and completed in accordance with the guidelines from the Animal Research and Ethics Board at McMaster University. Immunogenicity was assessed utilizing a well characterized candidate VSV-vectored vaccine expressing an *Mycobacterium tuberculosis *(*M.tb*) immune-dominant antigen, Ag85A (VSVAg85A). Given that VSV works best as a boosting vector^1^, all animals were primed initially with a human serotype 5 adenoviral (AdHu5) vector expressing Ag85A (AdHu5Ag85A). Immunization was carried out via the intramuscular route into the hind legs of the animal. All animals were prime-immunized with 1 $\times$ 10^7^ PFU of AdHu5Ag85A in a total volume of 100 µL of PBS (50 µL per hind leg). Two weeks following priming, animals were intramuscularly boost-immunized with either fresh liquid control of VSVAg85A (1 $\times$ 10^7^ PFU), stored liquid control (final PFU dose of 1 $\times$ 10^7^ PFU stored for 7 or 15 days at 37 °C), or reconstituted spray dried powders (viral vector concentration was adjusted to reach a final dose of 1 $\times$ 10^7^ PFU to account for spray dry process losses, as determined by in vitro testing) stored for 0, 7 or 15 days at 37 °C. Powder samples of the spray dried vaccine were reconstituted in 100 µL of sterile PBS solution. Control samples of the vaccines were completed in 100 µL of sterile PBS solution. Statistical analysis includes an unpaired *T*-tests comparing the liquid control to the spray dried vector at the three time points, where ***P = 0.0002 and ns: not significant.

### Spleen and lung mononuclear cell isolation

Mice were anesthetized by isoflurane administration prior to being sacrificed by cervical dislocation. Spleen mononuclear cells were isolated as described previously^19^. Briefly, the digested tissue was crushed through a 100 µm basket filter to obtain a single-cell suspension. Following centrifugation at 1,500 rpm, red blood cells were eliminated by treatment with ACK solution for 2 min. Isolated mononuclear cells were isolated were re-suspended in a complete RPMI 2,640 (RPMI 1,640 supplemented with 10% fetal bovine serum, 1% penicillin–streptomycin, 1% l-glutamine).

### Tetramer immunostaining and flow cytometry

Mononuclear cells from the spleen were immunostained and analyzed as previously described^19^. Cells were plated into U-bottom 96-well plates at a concentration of 20 million cells per mL. The cells were then washed and blocked with anti-CD16/CD32 in 0.5% bovine serum albumin/phosphate-buffered saline for 15 min on ice and then stained with the respective fluorochrome-labelled monoclonal antibodies. Cells were then processed according the manufacturer’s instructions (BD Biosciences, San Jose, CA). The monoclonal antibodies used included CD4-allophycocyanin-Cy7, CD8a-phycoerythrin-Cy7, and CD3-V450. For tetramer immunostaining, a tetramer for the immunodominant CD8 T-cell peptide (MPVGGQSSF) of Ag85A bound to the BALB/c major histocompatibility complex class I allele H-2L (NIH Tetramer Core, Atlanta, GA) was used. Immunostained cells were run on an LSR II flow cytometer (BD Biosciences, San Jose, CA) and 250,000 events per sample were collected and analyzed on the FlowJo software (version 10; Tree star, Ashland, OR).
